# Physical Activity Behavior Before, During, and After COVID-19 Restrictions: Longitudinal Smartphone-Tracking Study of Adults in the United Kingdom

**DOI:** 10.2196/23701

**Published:** 2021-02-03

**Authors:** Hannah McCarthy, Henry W W Potts, Abigail Fisher

**Affiliations:** 1 University College London London United Kingdom; 2 Institute of Health Informatics University College London London United Kingdom; 3 Department of Behavioural Science and Health University College London London United Kingdom

**Keywords:** physical activity, mobile apps, apps, fitness trackers, mHealth, COVID-19, behavior, tracking, smartphone, pattern

## Abstract

**Background:**

The COVID-19 pandemic led to the implementation of worldwide restrictive measures to reduce social contact and viral spread. These measures have been reported to have a negative effect on physical activity (PA). Studies of PA during the pandemic have primarily used self-reported data. The single academic study that used tracked data did not report on demographics.

**Objective:**

This study aimed to explore patterns of smartphone-tracked activity before, during, and immediately after lockdown in the United Kingdom, and examine differences by sociodemographic characteristics and prior levels of PA.

**Methods:**

Tracked longitudinal weekly minutes of PA were captured using the BetterPoints smartphone app between January and June 2020. Data were plotted by week, demographics, and activity levels at baseline. Nonparametric tests of difference were used to assess mean and median weekly minutes of activity at significant points before and during the lockdown, and as the lockdown was eased. Changes over time by demographics (age, gender, Index of Multiple Deprivation, baseline activity levels) were examined using generalized estimating equations (GEEs).

**Results:**

There were 5395 users with a mean age of 41 years (SD 12) and 61% (n=3274) were female. At baseline, 26% (n=1422) of users were inactive, 23% (n=1240) were fairly active, and 51% (n=2733) were active. There was a relatively even spread across deprivation deciles (31% [n=1693] in the least deprived deciles and 23% in the most [n=1261]). We found significant changes in PA from the week before the first case of COVID-19 was announced (baseline) to the week that social distancing restrictions were relaxed (Friedman test: χ^2^_2_=2331, *P*<.001). By the first full week of lockdown, the median change in PA was 57 minutes less than baseline. This represents a 37% reduction in weekly minutes of PA. Overall, 63% of people decreased their level of activity between baseline and the first week of COVID-19 restrictions. Younger people showed more PA before lockdown but the least PA after lockdown. In contrast, those aged >65 years appeared to remain more active throughout and increased their activity levels as soon as lockdown was eased. Levels of PA among those classed as active at baseline showed a larger drop compared with those considered to be fairly active or inactive. Socioeconomic group and gender did not appear to be associated with changes in PA.

**Conclusions:**

Our tracked PA data suggests a significant drop in PA during the United Kingdom’s COVID-19 lockdown. Significant differences by age group and prior PA levels suggests that the government’s response to COVID-19 needs to be sensitive to these individual differences and the government should react accordingly. Specifically, it should consider the impact on younger age groups, encourage everyone to increase their PA, and not assume that people will recover prior levels of PA on their own.

## Introduction

It is well established that sufficient physical activity (PA) is important for good health [1,2]. PA substantially reduces risks of common noncommunicable diseases including cardiovascular disease, diabetes, some cancers, and depression [1]. However, the COVID-19 pandemic resulted in worldwide implementations of restrictive measures to reduce social contact and viral spread. These varied by country, but have generally involved restrictions on nonessential movement and are likely to have had an impact on PA levels. Several papers have expressed concern about the negative consequences of reduced PA during these restrictions and the value of maintaining PA [3-6].

In the United Kingdom, throughout the COVID-19 lockdown, people were allowed to spend time outside for PA, with the exception of those who were “shielding” or those self-isolating due to COVID-19 exposure or symptoms. From March 23, 2020, to May 8 (Wales), May 11 (Scotland), or May 13 (England), individuals were allowed out for exercise once per day, respectively; subsequently, this changed to as often as was desired. This contrasted with more restrictive lockdowns seen in other countries, like Spain or France, where leaving home for exercise was not permitted. The inclusion of daily exercise in the United Kingdom lockdown guidance could account for the Sport England findings that 62% of 2000 people surveyed said they felt exercise was more important than before COVID-19 [7].

Evidence on the impact of pandemic restrictions on PA behavior varies and there are multiple methodological approaches available, each with strengths and limitations. Google Trends data showed that interest in exercise in April 2020 was higher than at any other time since records began. However, these data cannot tell us whether this increased interest was among those already habitually active or whether it translated into behavior change [8].

Cross-sectional surveys have been most commonly employed and have generally shown substantial declines in PA. The ECLB-COVID19 international survey gathered data from 1047 respondents from Africa (40%), Asia (36%), Europe (21%), and elsewhere (3%) April 6-11, 2020. They found substantial drops in PA in response to COVID-19 restrictions (eg, a 24% drop in the number of days/week of moderate-intensity PA and a 34% drop in the number of minutes of walking per day) [9]. Rogers and colleagues [10] conducted an online survey April 6-22, 2020, that found 25% of respondents reported doing less PA, while 12% reported doing more. Predictors of doing less PA were being female, not having access to a garden, having various pre-existing conditions, and expressing sentiments about personal or household risks. Older people (aged >70 years) were more likely to be doing the same intensity level of PA. People aged 20-34 were significantly more likely to have changed their PA levels to be either more or less intense than prior to the lockdown. More positive results were reported in a survey starting March 17, 2020, with 75% of respondents meeting PA guidelines. Meeting guidelines was associated with being female, being aged ≥65 years, having higher household income, and having had higher prior levels of PA, but negatively associated with prior physical symptoms [11].

All these surveys, which only captured the very early phases of the pandemic, used convenience sampling, using social media and snowballing approaches for recruitment. These approaches, while fast and low cost, can lead to sampling biases. In addition, it is very hard for individuals to accurately recall weekly minutes of PA even within recent timeframes (such as the last 7 days), let alone recalling PA prior to COVID-19 restrictions. Thus, these surveys are missing a reliable baseline measure. However, in recent years, many people have started routinely tracking their PA through the use of apps and wearables. This gives us a source of data that is longitudinal, predating the pandemic, and not reliant on self-report/recall.

One of the most widely used such technologies is the Fitbit; a blog by Fitbit described declines in PA in the week ending March 22, 2020 [12], including a 9% decline in step activity in the United Kingdom. All 20 countries studied showed declines, with the largest being a 38% decline in Spain, which had a more restrictive lockdown. By June 2020, Fitbit reported step count levels increasing but not yet back to the same level as last year, except among older women (aged 50-64 years), who surpassed the previous year’s levels. Younger Fitbit users seemed to be making the smallest step count gains. Fitbit differentiates between steps and active minutes, which are defined as more “vigorous and intentional” activity that is important for heart health. Vigorous activity had increased during the lockdown, but appeared to be returning to the same levels as last year across all age groups [13]. Similar trends were seen among users of Garmin fitness trackers, showing a reduction in steps and an increase in more vigorous PA [14]. Neither data set has been described in academic reports.

The largest tracking study to date had walking data from 455,404 users of a smartphone app in 187 countries and found a 27% decrease in steps between baseline and a month after the announcement of COVID-19 as a pandemic [15]. No demographic data were available, meaning it was not possible to characterize users or explore sociodemographic patterns.

Therefore, the aims of this study were to explore patterns of tracked activity (ie, walking, running, and cycling) in the United Kingdom before, during, and immediately after the COVID-19 restrictions were in place and to explore variations by demographic characteristics.

## Methods

### Overview

Participants were individuals in the United Kingdom registered with BetterPoints. BetterPoints is a free, publicly available, smartphone-based program that offers rewards (points, lottery style tickets, and virtual rewards such as medals) for the amount of PA tracked per week. Points can ultimately be converted to financial rewards (exact amounts and types are dependent on the program sponsor). Program sponsors include local government/councils, National Health Service trusts, Clinical Commissioning Groups, Development Corporations and, increasingly, large corporate entities. Further information about BetterPoints can be found on their website [16].

Individuals must be aged ≥14 years to register with the program. Registered users who had tracked any PA at all between January 22 and June 17, 2020, were included in the study. On registration with the app, users are asked to provide year of birth (used to derive approximate age), gender (male/female/other), and home post code (used to derive the Index of Multiple Deprivation [IMD] decile via the UK Government website [17]).

This study was approved by the UCL ethics committee (ID 401.001). When registering with the BetterPoints program, individuals agree and consent to the Terms and Conditions and Privacy Statement, including that their tracked data will be used to monitor patterns and that their “anonymized data may be shared with trusted non-BetterPoints entities to do research.”

PA was tracked by the BetterPoints smartphone app. Users could track their activities via a menu where they select activity types such as walking, running, or cycling, or they could turn on automatic tracking. From March 2020, automatic tracking became the default. BetterPoints uses proprietary algorithms to combine data from the chipsets in the phone (motion sensors, accelerometers, built-in classifiers) with additional data pertaining to speed, global positioning system data, and various map data sources to classify activity types automatically. The BetterPoints system records 0 if no valid activity is tracked. This is designed to avoid categorizing small amounts of movement (eg, walking around the house). The person must move over a distance, at a certain speed and acceleration, for the movement to qualify as walking, running, or cycling. To run automatic tracking, the smartphone must have a motion co-processor or accelerometer that monitors movement. Most current smartphones support this automatic tracking, including the Apple iPhone 5S (iOS) or above and nearly all Android-based phones. Smartphone sensors in iOS and Android phones have been shown to provide valid estimates of PA in naturalistic settings compared to ActiGraph [18] and pedometers [19].

The BetterPoints app displays data in a dashboard view of total weekly minutes of PA, which incorporates time spent walking, running, and cycling.

### Analysis

This was a retrospective study design using existing data in the context of a constantly evolving pandemic response, so pragmatic decisions had to be made, including about how to define a baseline period and which follow-up measurement periods should be included in some analyses. Key dates in the United Kingdom’s COVID-19 pandemic response were chosen, within the context of the data having a resolution of one week and attempting to reduce the number of unnecessary post hoc statistical tests. A summary of our measurement dates is provided in Table 1. Our weeks run from Wednesday to Tuesday because our data set began January 1, 2020, which was a Wednesday. The week commencing January 22, 2020, was selected as the baseline. This was the week before the first case of COVID-19 was reported in the United Kingdom.

**Table 1:** Baseline and follow-up measurement dates for analysis.

Date (week commencing)	Significant events
January 22	Study baseline (the week before the first COVID-19 case in the United Kingdom was announced)
March 11	Nonessential travel banned and social distancing introduced
March 18	The lockdown began on March 23
March 25	First full week of the lockdown
May 13	The lockdown was relaxed (multiple excursions for exercise allowed)
June 17	First full week with nonessential shops reopened (reopened on June 15)

Demographics were summarized using descriptive statistics. Participants were grouped into Sport England Active Lives’ categories of active (≥150 minutes of PA per week), fairly active (30-149 minutes), and inactive (0-29 minutes), according to the minutes of activity participants engaged in during the baseline week (commencing January 22, 2020) [20].

PA data were highly skewed, with many zero values (39% of all data values), so data were analyzed in three ways. First, median and mean PA were plotted by week, overall and median by demographics and activity levels at baseline. Next, nonparametric tests were used to compare PA over time. We performed a Friedman test to determine if there were significant differences in activity over time, then Wilcoxon tests were conducted to compare key follow-up weeks to baseline. Change in PA was calculated by computing baseline minus follow-up week. The mean, median, and interquartile range of this change score were then calculated. The change was also expressed as a percentage of baseline. Change categories were used to describe the proportion of people who had decreased, increased, or maintained their activity levels from baseline to the first full week of COVID-19 restrictions. These analyses were conducted in SPSS (Version 26, IBM Corp).

Finally, parametric tests were performed; specifically, generalized estimating equations (GEEs), a further generalization of the generalized linear model (GLM), were used to take into account the variance structure of the outcome data being from the same individuals over multiple weeks. We wanted to use a negative binomial regression, appropriate for the highly skewed data [21], but the GEE models failed to converge, presumably the result of a large number of zero values and high correlations between each week and the next. Instead, additional change scores were created. We calculated 20 sets of values using the amount of PA in each week minus the previous week for that individual. This change score is more amenable to analysis, producing a symmetric distribution (skew=–0.1). However, it is very leptokurtic (kurtosis=17.9). We carried out a modified square root transformation as shown in equation 1:

transformed value = sign(x) × √(|x|) (**1**)

This reduces the length of the data distribution’s tails and the kurtosis (now 3.3). We were then able to fit a linear regression GEE to the data. We used Huber-White sandwich robust variance estimators. These analyses were conducted in Stata (Version 11, StataCorp).

## Results

### Demographics

In total, 5395 users registered at least some activity each week from the week commencing January 22 to June 17, 2020, and were included in the current analysis. Participant characteristics are provided in Table 2. There were 130 missing values for gender data. In addition, 15 cases of approximate age >100 years were recoded as missing. Since there was no missing data for the primary outcome (PA) and <4% missing data for age or IMD, the decision was made not to impute these values. Users were on average 41 years old (SD 12; range 14-93 years). In addition, 61% of users identified as female, 37% as male, and 0.4% as other. There was a relatively even spread across deprivation deciles, with 31% falling in the least deprived deciles, and 23% in the most. At baseline, 26% of users were inactive, 23% were fairly active, and 51% were active.

**Table 2:** Participant characteristics and proportions of missing data (N=5395).

Characteristics		Values
Age (years), mean (SD)^a^		41.02 (12.2)
**Age categories (years), n (%)**		
	14-24	463 (8.6)
	25-34	1210 (22.4)
	35-44	1554 (28.8)
	45-54	1246 (23.1)
	55-64	567 (10.5)
	≥65	168 (3.1)
**Gender, n (%)**		
	Male	1971 (36.5)
	Female	3274 (60.7)
	Other	20 (0.4)
**Index of Multiple Deprivation, n (%)**		
	1-3 (most deprived)	1261 (23.4)
	4-7	2240 (41.5)
	8-10 (least deprived)	1693 (31.4)
	Missing	201 (3.7)
**Baseline physical activity, n (%)**		
	Inactive	1422 (26.4)
	Fairly active	1240 (23.0)
	Active	2733 (50.7)

^a^There were 187 missing values.

### Plots and Nonparametric Tests

**Figure 1:**
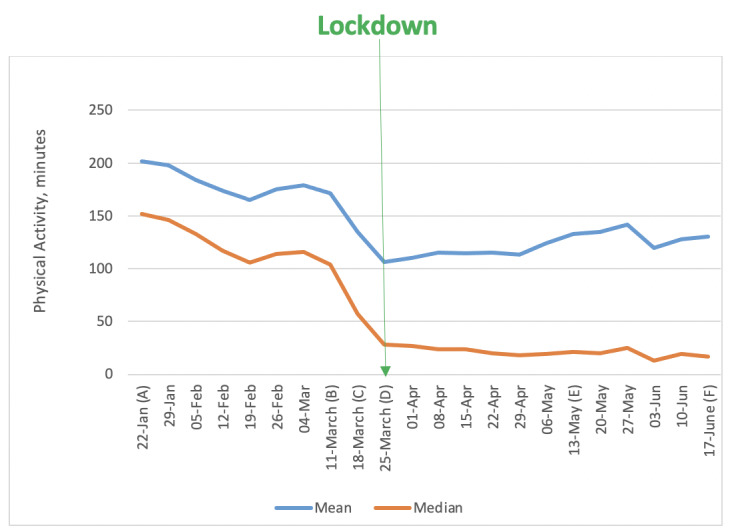
Weekly minutes of physical activity (mean and median). Important dates are marked on the x-axis as follows. (A) January 22: Baseline, week before first COVID-19 case announced in the United Kingdom. (B) March 11: Social distancing measures introduced. (C) March 18: Lockdown begins. (D) March 25: First full week of lockdown. (E) May 13: Lockdown measures relaxed. (F) June 17: Shops reopen.

**Figure 2:**
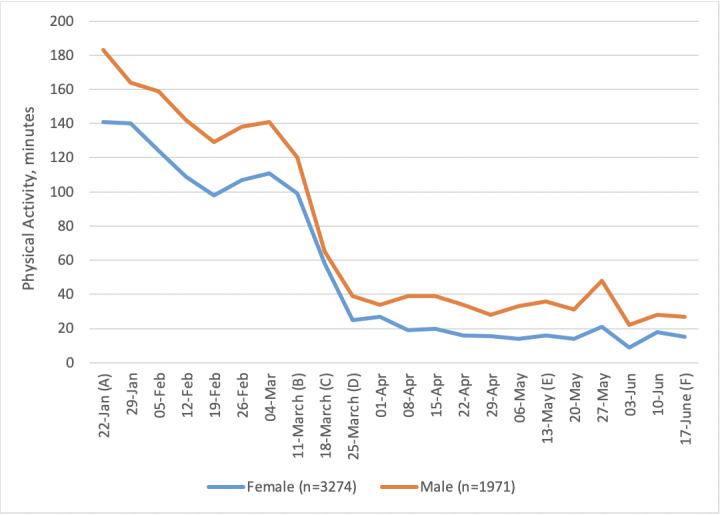
Weekly minutes (median) of activity in males and females (the "other" group was too small). Important dates are marked on the x-axis as follows. (A) January 22: Baseline, week before first COVID-19 case announced in the United Kingdom. (B) March 11: Social distancing measures introduced. (C) March 18: Lockdown begins. (D) March 25: First full week of lockdown. (E) May 13: Lockdown measures relaxed. (F) June 17: Shops reopen.

**Figure 3:**
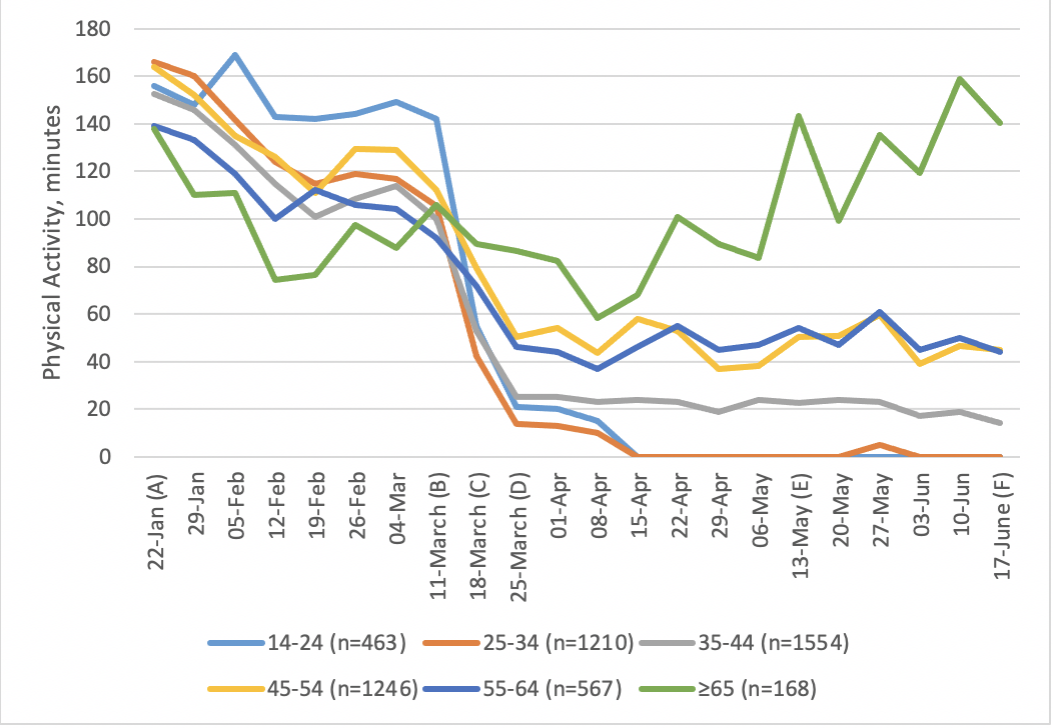
Weekly minutes (median) of activity by age. Important dates are marked on the x-axis as follows. (A) January 22: Baseline, week before first COVID-19 case announced in the United Kingdom. (B) March 11: Social distancing measures introduced. (C) March 18: Lockdown begins. (D) March 25: First full week of lockdown. (E) May 13: Lockdown measures relaxed. (F) June 17: Shops reopen.

**Figure 4:**
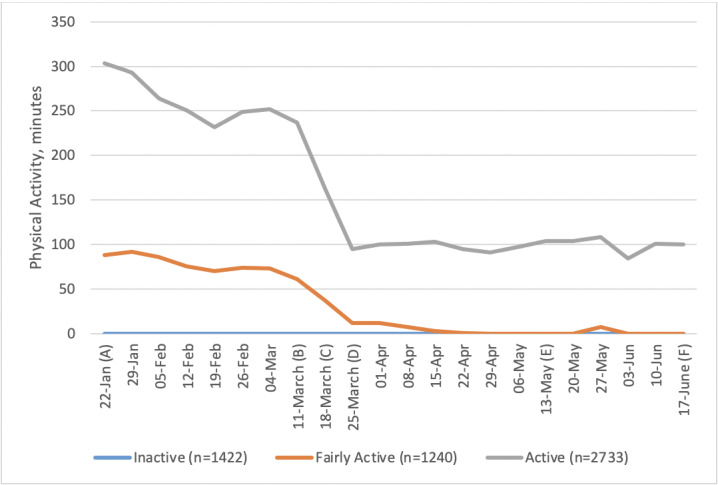
Weekly minutes (median) of activity by physical activity level at baseline. Important dates are marked on the x-axis as follows. (A) January 22: Baseline, week before first COVID-19 case announced in the United Kingdom. (B) March 11: Social distancing measures introduced. (C) March 18: Lockdown begins. (D) March 25: First full week of lockdown. (E) May 13: Lockdown measures relaxed. (F) June 17: Shops reopen.

**Figure 5:**
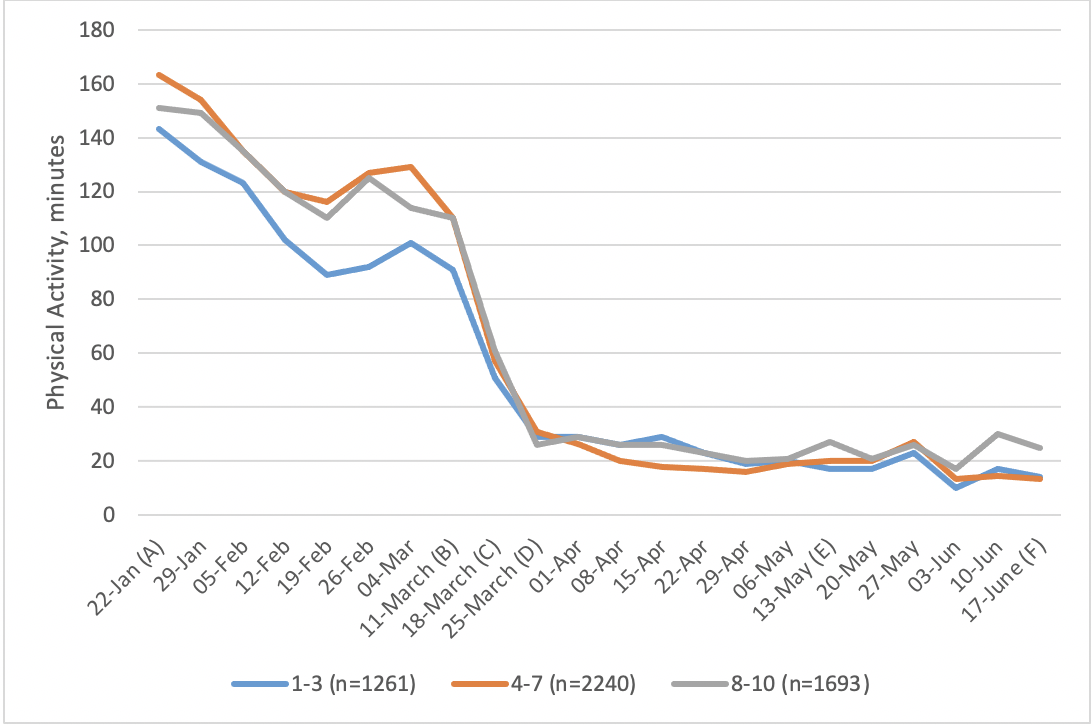
Weekly minutes (median) of activity by indices of multiple deprivation. Important dates are marked on the x-axis as follows. (A) January 22: Baseline, week before first COVID-19 case announced in the United Kingdom. (B) March 11: Social distancing measures introduced. (C) March 18: Lockdown begins. (D) March 25: First full week of lockdown. (E) May 13: Lockdown measures relaxed. (F) June 17: Shops reopen.

Plots of PA over the study period are shown in Figures 1-5. PA started to decline the week commencing March 11, 2020, when nonessential travel and social distancing measures were introduced. This decline continued until the first week of full lockdown, after which it appeared to remain fairly static throughout most of the lockdown, apart from a blip for the week commencing June 3, which had inclement weather.

Median weekly minutes of PA and the results of statistical tests of difference are presented in Table 3. There were significant differences in PA between the week before the first case of COVID-19 was announced (baseline) and the week that social distancing restrictions were relaxed (Friedman test: χ^2^_2_=2331, *P*<.001).

**Table 3:** Summary of physical activity at baseline, through lockdown, and beyond (January 22-June 17, 2020; N=5395).

Time points	Physical activity (minutes), median (IQR)	Friedman test
Baseline	152 (20-306)	N/A^a^
Social distancing introduced (March 11)	104 (0-273)	N/A
Lockdown begins (March 18)	57 (0-209)	N/A
First full week of Lockdown (March 25)	28 (0-158)	N/A
Lockdown relaxed (May 13)	21 (0-199)	N/A
Shops reopen (June 17)	17 (0-197)	χ^2^_2_=2331, *P*<.001

^a^N/A: not applicable.

There were statistically significant changes in weekly minutes of tracked PA at all time points leading up to lockdown and in the weeks following the easing of lockdown measures (Table 4). There was a 2-minute reduction in activity between the baseline week (January 22, 2020) and the week nonessential travel and social distance restrictions were introduced. The week that the lockdown was announced, PA dropped by more than 30 minutes. By the following week (the first full week of lockdown restrictions), PA was down by 57 minutes compared with baseline. The drop in PA from baseline to the lockdown represents a 37% reduction in PA.

**Table 4:** Wilcoxon tests of change in physical activity from baseline to key weeks during the COVID-19 restrictions (January 22-June 17, 2020).

Key events (week commencing)	Mean change (minutes)	Percentage decrease from baseline	Median change (minutes)	Percentage decrease from baseline	*P* value
Social distancing introduced (March 11)	30	15	2	1	<.001
The lockdown begins (March 18)	67	33	32	21	<.001
First full week of the lockdown (March 25)	95	47	57	37	<.001
The lockdown is relaxed (May 13)	69	34	42	28	<.001
General shops reopen (June 17)	71	35	41	27	<.001

Overall, 63% of people decreased their level of activity between baseline and the first week of COVID-19 restrictions, 16% of people did not change their PA, and 21% increased their PA. The median change in PA was very similar for males and females (Figure 2). There appeared to be an effect of age, whereby younger people engaged in more PA before lockdown and the least amount of PA after lockdown (Figure 3). In contrast, those aged ≥65 years appeared to remain more active throughout and increased their activity levels as soon as the lockdown was eased.

Levels of PA among those classed as active at baseline showed a dramatic drop (Figure 4). Median levels of PA among those classed as fairly active at baseline also dropped but less dramatically. People who were inactive at baseline remained inactive throughout. Socioeconomic group did not appear to be associated with changes in PA (Figure 5).

### GEE Models

A linear GEE was fitted to the data, with transformed week-on-week change in PA as the dependent variable. We explored the independent variables of gender, approximate age (as a continuous variable), deprivation decile, baseline activity level (inactive, fairly active, or active), and week (as a categorical variable). Examination of the data suggested an autoregressive correlation structure was appropriate. We fitted an AR(1) structure [22]. The resulting coefficients do not have a simple interpretation given the use of transformed data, but positive values indicate week-on-week increases in PA, while negative values indicate week-on-week decreases. Larger coefficients indicate larger changes. Sensitivity analyses were carried out using different assumptions for the correlation structure and with alternative modelling approaches. Similar results were found.

A basic model was fitted with just week as an independent variable, included as a categorical variable. This was statistically significant (χ^2^_20_=1759, *P*<.001). We then tested a model with the additional independent variables of age, gender, baseline activity, and deprivation index. This was statistically significant (χ^2^_24_=2562, *P*<.001). A likelihood ratio test showed improved fit from the additional variables (χ^2^_4_=895, *P*<.001). This indicated that older individuals showed more week-on-week increases in PA; that those who were most active showed more week-on-week decreases; and a pattern of increases and decreases over time matching Figure 1. All of these effects are independent of each other. There was no statistically significant relationship between week-on-week change and either gender or deprivation index, as shown in Table 5.

**Table 5:** Statistical analysis.

Variable		Coefficient	*P* value
Age (per decade)		0.08	<.001
Gender		–0.01	.50
Baseline activity (3 levels)		–0.38	<.001
Deprivation index		0.00	.60
**Change from previous week to week n**			
	To week 2	0.84	<.001
	To week 3	0.61	.001
	To week 4	0.32	.07
	To week 5	1.10	<.001
	To week 6	0.54	.002
	To week 7	0.33	.06
	To week 8 (social distancing introduced)	–2.45	<.001
	To week 9 (the lockdown begins)	–1.90	<.001
	To week 10 (first full week of the lockdown)	0.55	.001
	To week 11	0.62	<.001
	To week 12	0.27	.10
	To week 13	0.20	.20
	To week 14	0.17	.30
	To week 15	0.97	<.001
	To week 16	0.94	<.001
	To week 17 (the lockdown is relaxed)	0.28	.09
	To week 18	0.83	<.001
	To week 19	–1.40	<.001
	To week 20	0.88	<.001
	To week 21	0.50	.002
	To week 22 (general shops reopen)	Baseline	Baseline

We additionally wanted to investigate whether the relationship with age varied over time. We simplified the time variable, dividing the weeks into three phases: weeks 1-7 (prelockdown), weeks 8-9 (the lockdown started), weeks 10-21 (the lockdown continued). A model using these phases as independent variables rather than individual weeks does not fit as well, but it fits well enough to allow the investigation of interaction effects. We tested the addition of two interaction terms for age in the “lockdown started” phase and the “lockdown continued” phase: these were statistically significant (likelihood ratio test, χ^2^_2_= 49, *P*<.001). This confirmed that older participants showed less decrease in PA when the lockdown began, and a greater increase in PA as the lockdown continued, confirming the interpretation of Figure 3.

## Discussion

This longitudinal study of tracked PA, before, during, and after COVID-19 restrictions, showed large decreases in PA. Significant decreases in PA were observed at all time points from the week that social distancing measures were introduced, throughout the lockdown and the week measures were relaxed. PA was still significantly lower by the week commencing June 17, when nonessential shops reopened. The week that the lockdown was announced, median PA was down by 30 minutes and by the first full week of the lockdown, it had reduced by nearly an hour a week (57 minutes). This drop in PA represents a 37% reduction in individuals’ weekly minutes of PA. Older people were significantly more likely to maintain and then increase their PA levels during the lockdown. Those who were most active to begin with showed the biggest falls in PA, but they had the furthest to fall. Although men showed more PA on average throughout, in our GEE model there was no effect of gender on the decline of PA. The deprivation index did not show any relationship with PA, although IMD is only an approximate measure of an individual’s socioeconomic status, and the lack of evidence here may not translate into a lack of important socioeconomic effects.

Decreases in our study were substantially larger than the 9% drop observed from UK Fitbit step data [12]. Fitbit data ended the week of March 22, which was just before the full lockdown restrictions were in place, and our data showed a 21% decrease in PA in the same week (week commencing March 18; our weeks ran Wednesday-Tuesday). Our smartphone-based measure may have overestimated the decline, since activity like incidental steps accumulated in the home or workplace would not have been captured. However, our study also showed greater decreases in PA than found in the large international tracking study by Tison and colleagues [15], who also using smartphone tracking. UK data from that study showed approximately a 10% decrease in mean steps from their prepandemic baseline level (mean daily steps from January 19 to March 11, 2020) to March 25, compared with a 37% decrease in tracked minutes in this study. It is not until the first week of April that percentage decreases in UK step counts converged with our findings, at around a 33% decrease from baseline, although our data included an estimate of time spent cycling as well as walking/running. The percentage drop in weekly PA in our findings is also in line with the international ECLB-COVID19 study, which found a reported 34% drop in the number of minutes of walking per day [9].

Overall, our findings are less optimistic than some studies of PA response to the pandemic that relied on data captured at the start of social restrictions. Increased interest in and intention to exercise have been reported elsewhere [8,20]. If such intentions existed in our sample, it appears they were only translated into action among older age groups. Those who were more active at baseline had the largest drops in PA and inactive people remained so throughout, contrary to findings that suggest a surge in PA during lockdown [10,11,23]. Perhaps of most concern are the fairly active group, who were close to doing recommended levels of PA for a number of weeks prior to lockdown and then dropped to doing no PA, with no sign of change as the lockdown was eased. We should not presume that these people will just automatically return to their prelockdown levels of exercise as restrictions are lifted. Measures to encourage this group back to prior levels of PA and continue to build on those efforts seem worthwhile.

In our study, older people showed less of a decrease in PA when the lockdown was introduced and recovered their PA levels during the lockdown faster than their younger counterparts. Concordant results were found by Fitbit [13] and by Smith and colleagues [11,24]. Our findings build on these prior results in suggesting that differences in maintained and increased PA in older age groups continued throughout the lockdown (and beyond). It is particularly interesting to note that approximately half of our sample of older adults were aged >70 years; that group had been urged by the UK Government not to go outside for even one daily bout of exercise. Although this is reassuring news about PA behavior in those aged >65 years, the large reduction in PA levels and failure to show any recovery for those below middle age is concerning. This picture suggests that policy interventions may be better focused on the long-term impact of restrictions on younger groups, which supports conclusions from recent research [11].

It was not possible to establish why we observed this pattern with age in our data. However, there is evidence that younger people are more worried about COVID-19, which may be a factor [25]. It is also feasible that factors like working/schooling from home had a stronger influence in younger age groups, but this is an area for future study.

Our study only tracked PA accumulated outdoors; some people may have substituted outdoor PA with indoor PA. BetterPoints users were given the opportunity to record activities such as “Be active at home” and “Try something new” during the lockdown, but analysis of this data was beyond the scope of the present analysis and may be included in future studies. The inclusion of live-streamed and prerecorded exercise classes in the app is currently being explored, while ways to routinely capture this type of activity and further surveying of users to understand whether they shifted to indoor exercise is underway. Wearables data suggest that while step counts may have reduced during the pandemic, vigorous activity may have increased, although these gains have not necessarily been maintained as we emerged from the lockdown [13]. This kind of activity may have continued among the previously active group but cannot be known from the current data.

While the approach of using app data gives us a good baseline and more valid measures of PA, we do have a sample bias as the results depend upon who uses BetterPoints. It may be that older adults who are using the BetterPoints app are a particular group who are more interested in exercise in the first place. Other limitations inherent in using data collected from smartphones include variability in how people carry and interact with their phones and variability in how many people track data on any given day. Although data on age, gender, and IMD were collected, information on other important demographics (like ethnicity) were not. In addition, although the app is free to use, smartphone ownership may be less common in some groups (eg, those with very low income). Only 79% of the UK population has a smartphone (2019 data), which means we cannot be sure how representative our data are of the UK population [26].

We have shown that tracked PA data suggests larger drops in PA during the United Kingdom’s COVID-19 lockdown than indicated previously. Significant differences by age group and prior PA levels suggests that blanket conclusions cannot be drawn about the impact of social distancing measures on population PA. The importance of better understanding in how to engage with and support different groups in tailored ways cannot be underestimated. Government response to COVID-19, particularly during the current situation where renewed outbreaks lead to local restrictions being imposed, needs to be sensitive to these individual differences and the government must react accordingly.

## Abbreviations

GEE: generalized estimating equation
GLM: generalized linear model
PA: physical activity
